# [Expression of Concern] NIMA-related kinase 2 regulates hepatocellular carcinoma cell growth and proliferation

**DOI:** 10.3892/ol.2025.15226

**Published:** 2025-08-13

**Authors:** Xiao-Bo Lai, Yu-Qiang Nie, Hong-Li Huang, Ying-Fei Li, Chuang-Yu Cao, Hui Yang, Bo Shen, Zhi-Qiang Feng

**Affiliations:** Department of Gastroenterology and Hepatology, The First Municipal People's Hospital of Guangzhou, Guangzhou Medical University, Guangzhou, Guangdong 510180, P.R. China; Department of Gastroenterology, The Second Affiliated Hospital, Guangzhou Medical University, Guangzhou, Guangdong 510282, P.R. China

Oncol Lett 13: 1587–1594, 2017; DOI: 10.3892/ol.2017.5618

Following the publication of this paper, it was drawn to the Editor's attention by a concerned reader that, regarding the flow cytometric plots shown in Fig. 4B, the “Blank” and “NC” panels looked unexpectedly similar, where the results from differently performed experiments were intended to have been portrayed; similarly, for the western blot data shown in Fig. 5, the Nek2 and CycE protein bands looked unexpectedly similar, suggesting that the same data had been included twice in this figure.

The authors were contacted by the Editorial Office to offer an explanation for these apparent anomalies in the presentation of the data in this paper; however, up to this time, no response from them has been forthcoming. Owing to the fact that the Editorial Office has been made aware of potential issues surrounding the scientific integrity of this paper, we are issuing an Expression of Concern to notify readers of this potential problem while the Editorial Office continues to investigate this matter further.

